# mRNA-Seq reveals the quorum sensing system *luxS* gene contributes to the environmental fitness of *Streptococcus suis type* 2

**DOI:** 10.1186/s12866-021-02170-w

**Published:** 2021-04-13

**Authors:** Jinpeng Li, Yuxin Wang, Yanbin Du, Hui Zhang, Qingying Fan, Liyun Sun, Li Yi, Shaohui Wang, Yang Wang

**Affiliations:** 1 College of Animal Science and Technology, Henan University of Science and Technology, Luoyang, China; 2 Key Laboratory of Molecular Pathogen and Immunology of Animal of Luoyang, Luoyang, China; 3 China Animal Health and Epidemiology Center, Qingdao, China; 4 College of Life Science, Luoyang Normal University, Luoyang, China; 5 Shanghai Veterinary Research Institute, Chinese Academy of Agricultural Sciences, Shanghai, China

**Keywords:** *Streptococcus suis*, *luxS* gene, Acid resistance, Iron stress, Oxidative stress

## Abstract

**Background:**

*Streptococcus suis type* 2 (SS2) is an important zoonotic pathogen. We have previously reported the structure of LuxS protein and found that the *luxS* gene is closely related to biofilm, virulence gene expression and drug resistance of SS2. However, the mechanism of *luxS* mediated SS2 stress response is unclear. Therefore, this experiment performed stress response to *luxS* mutant (Δ*luxS*) and complement strain (*C*Δ*luxS*), overexpression strain (*luxS*+) and wild-type SS2 strain HA9801, and analyzed the differential phenotypes in combination with transcriptome data.

**Results:**

The results indicate that the *luxS* gene deletion causes a wide range of phenotypic changes, including chain length. RNA sequencing identified 278 *lx*-regulated genes, of which 179 were up-regulated and 99 were down-regulated. Differential genes focus on bacterial growth, stress response, metabolic mechanisms and drug tolerance. Multiple mitotic genes were down-regulated; while the ABC transporter system genes, cobalamin /Fe^3+^-iron carrier ABC transporter ATPase and oxidative stress regulators were up-regulated. The inactivation of the *luxS* gene caused a significant reduction in the growth and survival in the acid (pH = 3.0, 4.0, 5.0) and iron (100 mM iron chelator 2,2′-dipyridyl) stress environments. However, the mutant strain Δ*luxS* showed increased antioxidant activity to H_2_O_2_ (58.8 mmol/L).

**Conclusions:**

The *luxS* gene in SS2 appears to play roles in iron metabolism and protective responses to acidic and oxidative environmental conditions.

**Supplementary Information:**

The online version contains supplementary material available at 10.1186/s12866-021-02170-w.

## Background

*Streptococcus suis* (SS) is a major pathogen in pigs, and it is also a zoonotic agent of a variety of diseases for swine and humans. Among the thirty-three serotypes of SS (serotypes 1–32 and serotype 1/2), *Streptococcus suis serotype 2* (SS2) are generally considered to be the most virulent serotypes found so far. It can cause a variety of life-threatening infections, including meningitis, arthritis and sepsis [1, 2]. The LuxS/AI-2 quorum sensing (QS) system is considered a process by which bacteria communicate using autoinducers 2 (AI-2). It is widespread in Gram-positive and Gram-negative bacteria. LuxS-mediated QS mechanism is based on the production of AI-2 that regulates various important biological properties in different bacteria [3]. In our previous study, we have shown that the loss of *luxS* gene can reduce the biofilm formation ability, hemolytic activity, adhesion to human laryngeal carcinoma (HEp-2) cell line and virulence genes transcription, and the *luxS* gene also related to drug-resistant efflux gene expression of SS [4–7]. Furthermore, our research found that *luxS* gene can regulate *pdh* genes that affect acid stress and oxidative stress of *S. suis* [8]. The ability of bacteria to resist environmental stress is one of the important factors for their survival. Studies have shown that LuxS protein is involved in regulating changes in bacterial resistance to stress [9]. However, the relevant mechanisms remain unclear.

The quorum sensing system is an important protective mechanism for bacteria to adapt to the environment [3, 10]. Previous studies have shown that *luxS* and AI-2 involved in bacterial regulation of a series of important stress responses, including heat shock, anti-gamma radiation, H_2_O_2_ and other oxidative stress responses [11–15]. With the deepening of *luxS* research, researchers found that *luxS* showed different phenotypes in some bacteria after mutation. For example, the *luxS* mutation in *Helicobacter pylori* leads to a decrease in the expression of flagella transcription regulator *flhA* and a decrease in motility [16]. Compared with the wild-type strain, the *luxS* mutant of *Escherichia coli* has increased survival in the environment with pH < 3.2 [17]. In addition, the ABC transporter gene of radiation *Deinococcus radiodrans luxS* mutant is up-regulated under oxidative stress, and ABC transporter participates in the adaptation mechanism of stress environment, transporting damaged nucleotides and polypeptides to vitro. And it’s proved that the *luxS* mutant of *Borrelia burgdorferi* has reduced pathogenicity in mice [18]. Moreover, Liu et al. found that overexpression of *luxS* gene could improve the stress resistance of *Lactobacillus paraplantarum* L-ZS9 [9]. We have found *luxS* gene as an important regulator in many aspects [10]. However, it is still unknown that the impact of the *luxS* genes on SS growth and stress responses. In order to understand the *luxS* gene more fully, in this study, we wanted to know the differences of wild-type strain, Δ*luxS*, *C*Δ*luxS* and *luxS*+ in growth characteristics and stress responses.

## Results

### Cell morphology

**Fig. 1:**
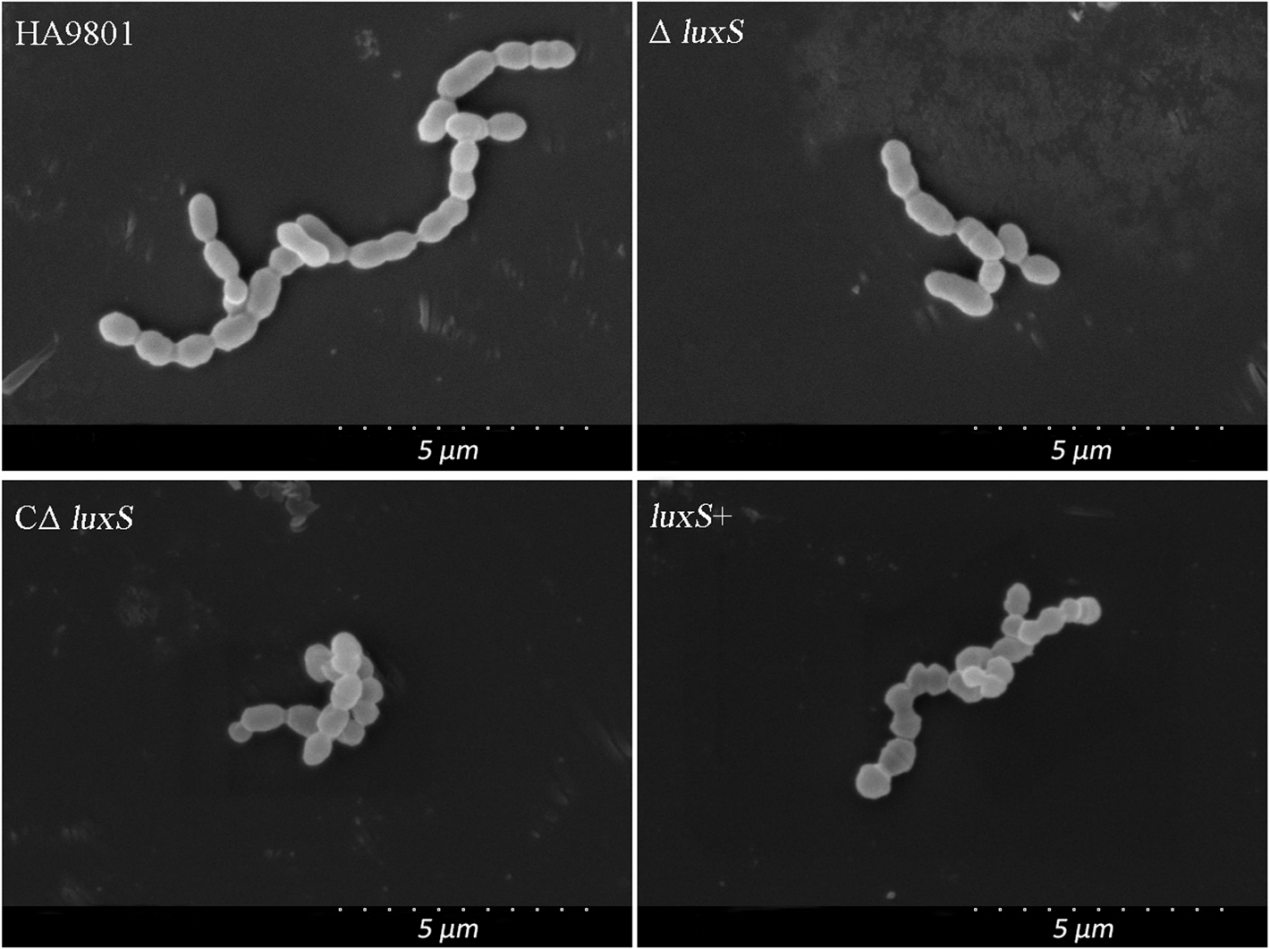
The morphology of SS2 wild-type strain HA9801, mutant strain Δ*luxS* complemented strain CΔ*luxS* and overexpression strain *luxS*+ under SEM

Through SEM observations, mutant strain Δ*luxS* tended to grow in chain length and exhibited abnormal morphology relative to the wild-type strain (Fig. 1). In addition, the aggregation ability of Δ*luxS* cells was significantly weaker than that of wild-type strain (Fig. S1). These morphology phenotypes can be restored in part by *C*Δ*luxS*. However, the differences between the overexpression strain *luxS+* and wild-type strain were not significant. Gram stain results showed that the morphological characteristics of the four strains consistent with the results of SEM (data not shown).

### Growth curves

The growth curves of wild-type, Δ*luxS*, *C*Δ*luxS* and *luxS*+ SS2 strains are presented in Fig. S2 (Supplementary material). Compared with the wild-type strain, the mutant strain Δ*luxS* did not show growth defects. For complementary strains, the difference between CΔ*luxS* and overexpression strain *luxS*+ is not significant (*P*>0.05).

### Δ*luxS* mutant and wild-type strain transcriptome analysis

**Fig. 2:**
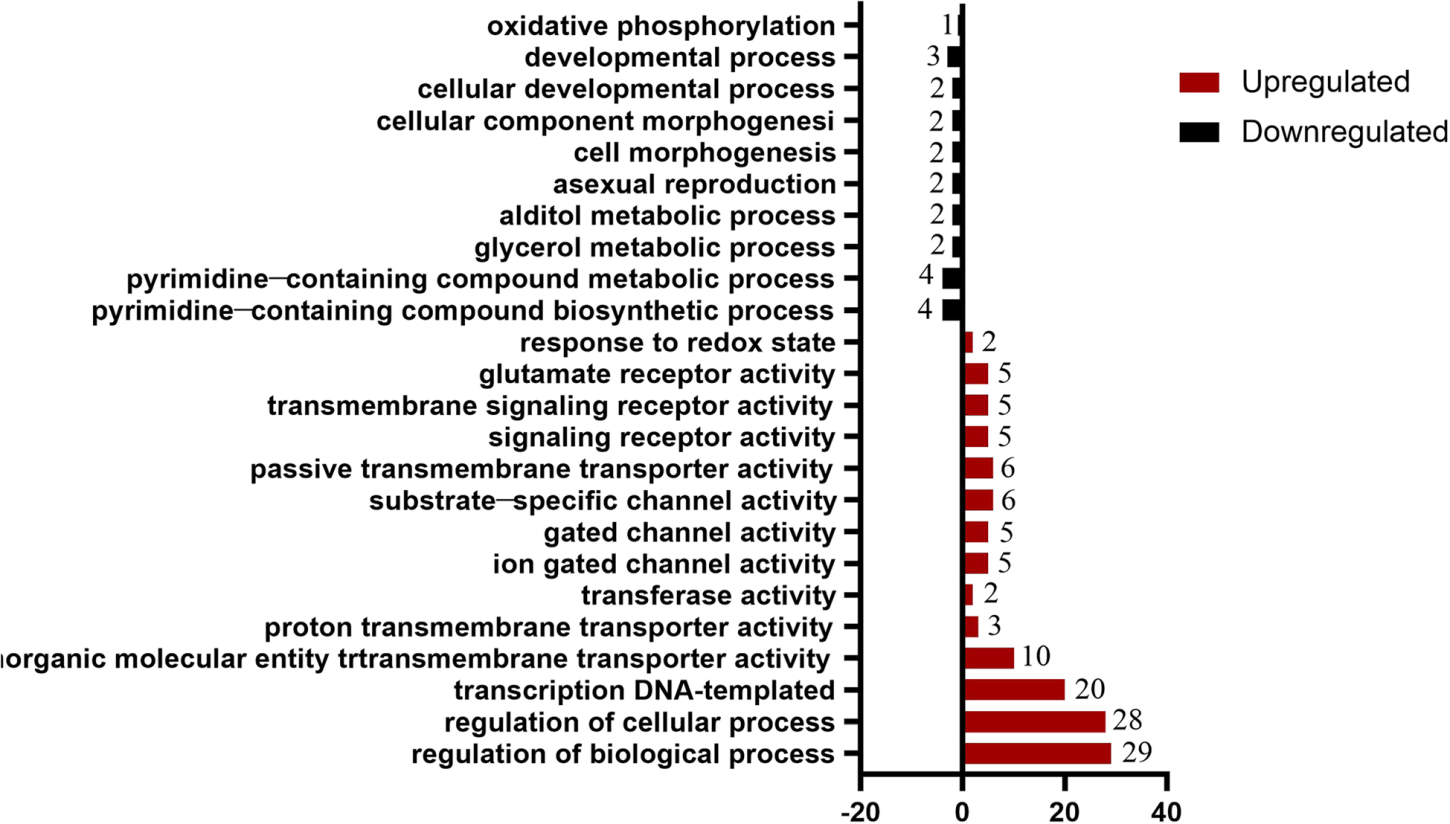
Transcriptome analysis of mutant strain Δ*luxS* compared to wild-type strain. Gene ontology analysis of differentially expressed genes. The red and black bars represent down-regulated and up-regulated genes, respectively, and the digital tags represent the number of genes associated with this pathway

Analysis of transcriptome data shows that *luxS* gene mutation has a wide-ranging effect on the gene expression of SS*.* There were 1978 identically expressed genes in the SS wild-type strain and the mutant strain Δ*luxS*. In addition, there were 179 up-regulated genes and 99 down-regulated genes (Table S1; Table S2, supplementary material). Obviously, the amino acid ABC transporter permease gene expression was up-regulated most and acetyltransferase gene, cell division genes was down-regulated most. Gene Ontology (GO) analysis identified the biological functions of differentially expressed genes, and found that the main enrichment of differential genes was in biological processing and molecular functions. There was no significant enrichment of differential genes in cellular components, as shown in Fig. 2.

### qRT-PCR

In order to verify the reliability of the RNA-seq results, 6 differentially expressed genes were randomly selected for verification (Fig. S3, supplementary material). The results showed that SSU05_2024, SSU05_1111, and SSU05_1069 were significantly up-regulated, and SSU05_0050, SSU05_0087, and SSU05_0302 were significantly down-regulated. The above results are consistent with the RNA-seq results (*P*>0.05), indicating the reliable of transcriptome results. In addition, qRT-PCR analysis was performed to compare WT, Δ*luxS*, CΔ*luxS* and *luxS*+ strains for the expression of several genes involved in environmental fitness. The expression of SSU05_1677, SSU05_0650, SSU05_2171, SSU05_1508 was significantly (*p* < 0.01) decreased and SSU05_1111, SSU05_1069 was significantly increased (Fig. S4). There was no significant difference between the WT, and CΔ*luxS* and *luxS*+.

### Acid tolerance of SS

**Fig. 3:**
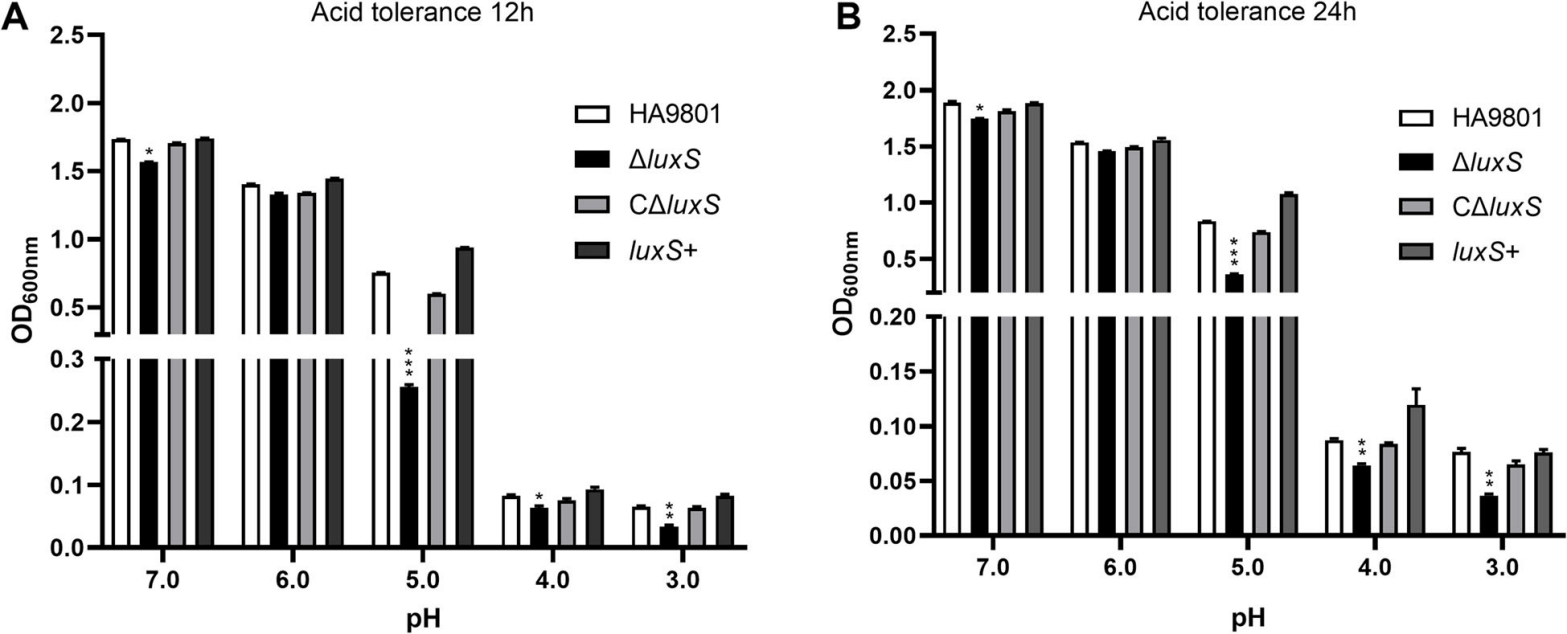
Acid resistance trends of SS2 strains at 12 and 24 h. The SS2 bacterial suspension was inoculated in THB liquid medium with pH values of 3.0, 4.0, 5.0, 6.0, 7.0 (adjusted with 6 N HCl), and the growth of each strain was measured at 12 h and 24 h. The growth and survival of mutant strain Δ*luxS* was significantly decreased in pH 3.0, 4.0 and 5.0 acidic environments compared with wild-type and complemented strain. The columns express the means and standard deviations of three or more experiments. *, significantly different at *p* < 0.05; **, significantly different at *p* < 0.01; ***, significantly different at *p* < 0.001

The acid tolerance assay suggested that four strains (HA9801, Δ*luxS*, CΔ*luxS*, and *luxS*+) viability decreased with pH value of the medium. Wherein, compared with pH values of 5.0, 6.0, 7.0, the OD_600nm_ values of the four strains at pH = 3.0 and pH = 4.0 decreased significantly. In addition, there is no difference in growth status among the HA9801, CΔ*luxS*, and *luxS*+ strains in acidic environments. However, the mutant strain Δ*luxS* showed significantly decreased survival (*P*<0.05) in acidic environments ranging from pH 3.0 to pH 5.0 at tested times compared with wild-type strain (Fig. 3), and the viable count of ΔluxS was lower than that of WT at PH = 5 for 12 h or 24 h (*P* < 0.001). Moreover, complementation of *luxS* gene restored the acid resistance for the complemented strain (*P*>0.05). These results indicated that *luxS* gene contributed to the acid tolerance of SS2.

### Fe stress response

**Fig. 4:**
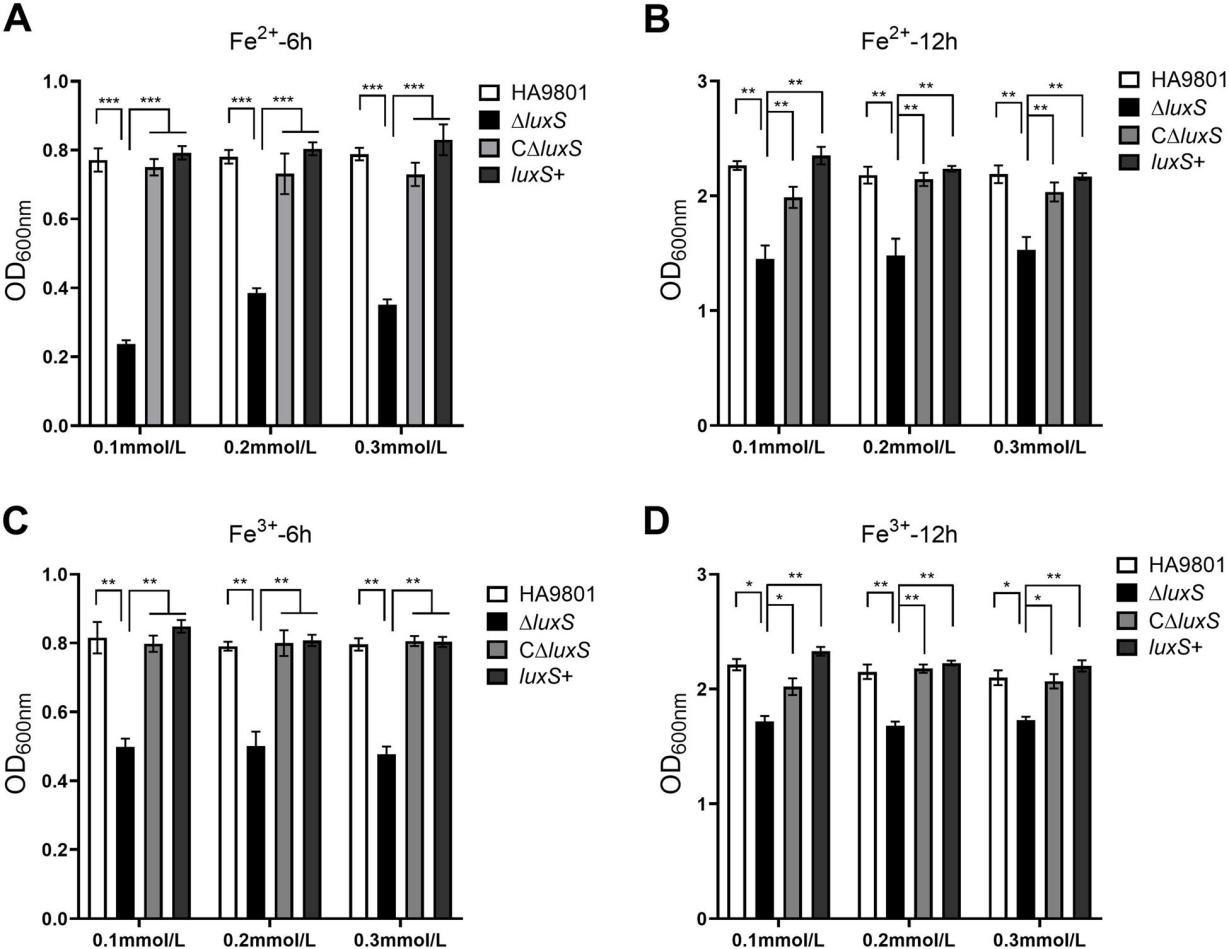
Effect of exogenous Fe^2+^ and Fe^3+^ on SS2 growth. The SS2 strain was inoculated on an iron-restricted medium (containing 100 mM iron chelator 2,2′-bipyridine), and added with different concentrations of iron ions, namely Fe^2+^ (0.1, 0.2 and 0.3 mmol / L) and Fe^3+^ (0.1, 0.2 and 0.3) mmol /L), and the growth of each strain was measured at 6 h and 12 h. The addition of exogenous Fe^2+^ and Fe^3+^ exerted a significant growth reducing effect on the mutant strain compared with wild-type, complemented and overexpression strains. The columns express the means and standard deviations of three or more experiments. *, significantly different at *p* < 0.05; **, significantly different at *p* < 0.01; ***, significantly different at *p* < 0.001

The effects of exogenous Fe^2+^ and Fe^3+^ on the growth of SS were determined. It was observed that wild-type and *luxS*+ strains, in the presence of 3 mmol/L iron chelator were not significantly impacted by the metabolic stressor (*P* > 0.05) at 6 h or 12 h. However, mutant strain Δ*luxS* was observed to have a significantly reduced OD_600nm_ value (*P* < 0.001) compared with wild-type strain. Moreover, the growth capacity of CΔ*luxS* was retored by the *luxS* gene complementation. The effect of Fe^2+^ on the growth of Δ*luxS* was more pronounced than that of Fe^3+^ (Fig. 4).

### Oxidative stress response

**Fig. 5:**
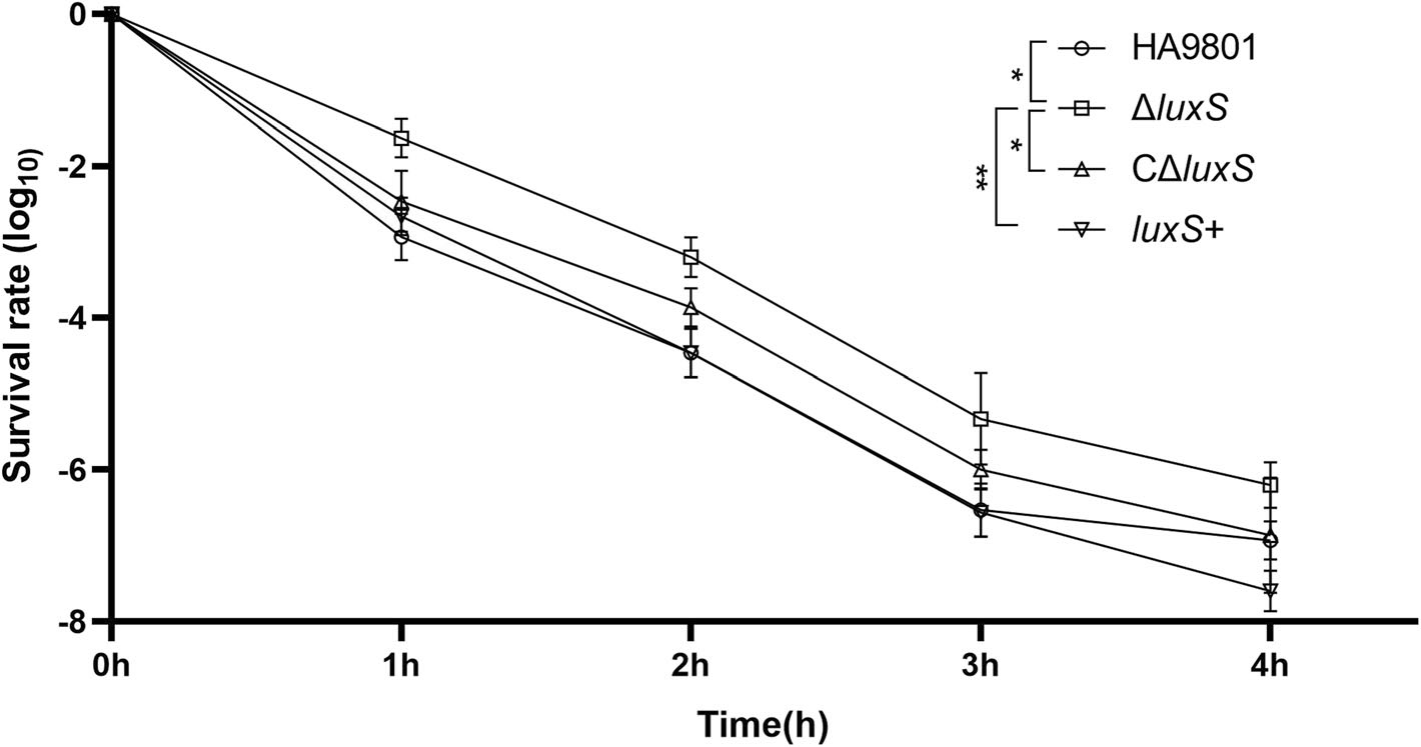
Survival rates of SS2 strains under oxidative stress. The SS2 strains were inoculated at medium containing 58.8 mmol/L H_2_O_2_, and the survival of each strain was measured at indicated time. The survival rate of mutant strain Δ*luxS* was higher than those of the wild-type strain, CΔ*luxS* and *luxS*+ at 1 h, 2 h, 3 h or 4 h. *, significantly different at *p* < 0.05; **, significantly different at *p* < 0.01; ***, significantly different at *p* < 0.001

To assess the capability of the *luxS* gene to manage oxidative stresses, survival of the SS2 wild-type, Δ*luxS*, *C*Δ*luxS* and *luxS*+ strains were measured after 1 h, 2 h, 3 h or 4 h of H_2_O_2_ treatment. The results indicated that the wild-type strain were more susceptible to H_2_O_2_ treatment (58.8 mmol/L) than the mutant strain Δ*luxS*. Survival rate of the *C*Δ*luxS* and wild-type strains were significantly different from that of Δ*luxS* (*P* < 0.05), and it was very significant with *luxS*+ strain (*P* < 0.01). Therefore, it can be concluded that *luxS* is associated with the antioxidant activity of SS2 (Fig. 5).

## Discussion

The highly conserved *luxS* gene has been extensively studied in recent years due to its involvement in the regulation of the expression of various growth and virulence-related genes [19, 20]. The related AI-2 is a compound that plays a key role in bacterial cell-to-cell communication [21]. In this study, we compared the cell morphology and response of mutant strain Δ*luxS* and wild-type strain HA9801 to different stress conditions. The transcriptome differences between HA9801 and Δ*luxS* were also determined. Our results showed that mutant strain Δ*luxS* showed a large transcriptional difference and significantly different tolerance to stress environments.

We found that the growth rate of mutant strain Δ*luxS* was similar with those of wild-type, CΔ*luxS* and *luxS*+ strains. This experimental results corroborate the findings of Zhang et al. [22] and Van et al. [23], in which the absence or overexpression of the *luxS* gene no affecting the expression of other downstream genes important for bacterial growth. Previous studies also reported that *luxS*+ did not increase the level of AI-2 production, and no affected the growth of SS [1].

Acid resistance is a necessary for bacterial survival in acidic environments and during infection of the host through the digestive tract. In the present experiment, the acid stress test revealed an overall downward trend in vitality in acidic environmental conditions. The downward trend of Δ*luxS* was more pronounced than that of wild-type strain, and the viability decreased precipitously when the pH < 5.0. Acid stress on *luxS*+ showed slightly stronger acid resistance than wild-type strain. The loss of the *luxS* gene results in many altered traits, including thinning of the bacterial capsule, which may be a cause of the altered acid resistance. In addition, with the down-regulated of the cell division protein genes SSU05_0760 and SSU05_0761, the Δ*luxS* strain showed abnormal cell chains. The results were similar to Cao et al. [24]. At same, previous studies have also found that *luxS* gene is involved in biofilm formation [10]. At pH = 3.0, the wild-type and Δ*luxS* strains had not yet been completely killed, suggesting that the *luxS* gene is part of a redundant system through which SS2 creates its acid resistance. These observation suggest that acid regulation is a complex process that may be influenced through a variety of regulatory pathways [25], the SS2 *luxS* gene appears to be part of one such regulatory pathway.

Iron is actually an essential element in all cells because iron is a cofactor in many enzymes, especially central metabolism and respiratory enzymes, so it is a coenzyme. Wen et al. [26] found that the growth inhibition of Δ*luxS* in the medium containing 0.3 mmol/L 2,2′-bipyridine was alleviated by adding exogenous iron and culture supernatant regulated by wild-type strains. In addition, Lee et al. [27] used to the gene chip to analyze and compare the transcription data of wild-type and mutant Δ*luxS* strains of *Streptococcus pneumoniae* at 4 different growth phages. It was found that two TonB systems are involved in iron absorption. Furthermore, tonB1-exbB1-exbD1 and tonB2-exbB2-exbD2 are affected by the *luxS* gene in all four growth stages [28]. Although nature is rich in iron, it is easily oxidized to insoluble matter, so the iron available to bacteria is scarce. Moreover, the easily soluble Fe tends to react with oxygen to produce reactive oxygen intermediates and affect the physiological processes of bacteria. Therefore, for *S. suis*, it is necessary to suppress the harmful effects of Fe and to store the excess Fe in a removable form. So iron transportability is very important. In the present study, the results showed that the addition of exogenous Fe^2+^ resulted in reduced growth of the wild-type strain with increasing ion concentration at 6 h and 12 h. The effects caused by Fe^3+^ were more pronounced than those of Fe^2+^ on growth promotion of the Δ*luxS* mutant strain, although the difference was not significant (*P* > 0.05). There was no significant difference observed in the growth status between wild-type and *luxS*+ strains (*P* > 0.05). In comparison to the wild-type and *luxS*+ strains, the growth of the Δ*luxS* mutant was observed to be significantly decreased (*P* < 0.001). The growth ability *C*Δ*luxS* did recover, albeit not to the level of the wild-type strain. Besides, in the mutant strain, the expression of the cobalamin / Fe^3+^-iron carrier ABC transporter ATPase gene SSU05_0650 was up-regulated, up to 1.976 times. We speculate that the reason why the mutant strain is more sensitive to Fe^2+^ than Fe^3+^ in the experiment may be due to the enhanced ability of the mutant strain to transport Fe^3+^ ions [29]. Taken together, the data suggest that the *luxS* gene is involved in SS iron absorption, and the regulation of bacterial growth.

Oxidative stress is one defense mechanism of the host against an invading pathogen. Bacterial infections must first overcome the host’s stress defense mechanism. Yu et al. [30] knocked out the *luxS* gene of *Yersinia pestis,* and observed that the mutant exhibited a reduced resistance to H_2_O_2_. However, the *Porphyromonas gingivalis* Δ*luxS* strain exhibited increased survival in the presence of H_2_O_2_ [31], this finding is similar to what was previously reported by Cao et al. [24] with respect to a H_2_O_2_ stress test of Δ*luxS* of SS2 strain 05ZYH33. The results of this experiment showed that mutant strain Δ*luxS* were more tolerant to H_2_O_2_ than the wild-type strain. This suggests that *luxS* genes have different roles in different bacterial species. After analyzing comprehensive transcriptome data, we believe that the differences in stress resistance of Δ*luxS* and wild-type strains may result in phenotypic differences due to the abnormal transcription of some genes after the *luxS* gene is mutant. Transcriptome data showed that the SS *spxA* transcriptional regulator SSU05_1111 and the possible oxidative stress-related gene SSU05_1069 were found in Δ*luxS* strains, and their expressions were up-regulated by 2.5 and 2.1 fold, respectively. It has a certain tolerance to H_2_O_2_ compared with the wild strain; research shows that *spxA* transcriptional regulators play an important role in oxidative stress response of SS. *spxA* mutant strains are more sensitive to the oxidative environment of SS [32, 33].

## Conclusion

In summary, the results presented here clearly demonstrate that there is a transcriptional difference between the SS2 WT strain and the Δ*luxS* strain. We also proved that the quorum sensing system *luxS* gene is of great significance to the morphological structure as well as stress resistance of SS2.

## Materials and methods

### Bacterial strain and culture conditions

Four *S. suis* strains were used in this study: the SS2 virulent wild-type strain HA9801 was isolated from pigs in the Jiangsu Province in 1998, and its mutant strain Δ*luxS*, complemented strain CΔ*luxS*, overexpression strain *luxS*+ of HA9801 was constructed in our previous study [1, 2]. The above four strains were preserved in our laboratory, and we verified the four strains by PCR before tests to ensure the correctness. The SS strains were grown at 37 °C in Todd Hewitt broth (THB) (Becton, Dickinson and Company, USA) medium or plated on THB agar with 5% (vol/vol) sheep blood (Becton, Dickinson and Company, USA).

### Morphological characteristics

The morphological differences were determined by scanning electron microscopy according to the method previously described [34]. Briefly, coverslips with SS2 cultures were rinsed 3 times with a phosphate buffered solution (Sigma-Aldrich, USA). The samples were then post-fixed for 90 min with 1% (w/v) osmium tetroxide (Hubei Baizhiang Biochemical Co., Ltd., China) in a 0.1 M sodium cacodylate buffer (Shanghai Xinyu Biological Technology Co., Ltd., China). After staining, the specimens were dehydrated in increasing concentrations of acetone (Shanghai Xinyu Biological Technology Co., Ltd., China) (10, 30, 50, 70, 90, and 100%). The specimens were then air-dried for 60 min, and were then adhered to metal holders with double-sided tape for coating with gold and palladium in an evaporator. All specimens were positioned with the apices facing up for proper visualization by scanning electron microscopy (SEM) in a vacuum at 5 kV electron beam energy (Hitachi S4700 FESEM; Hitachi Ltd., Tokyo, Japan). The bacterial morphology was also observed by gram staining and optical microscope (OM).

### Growth curve

The logarithmic growth phase SS2 cultures were diluted 1: 200 to achieve an optical density at 600 nm (OD_600nm_) of approximately 0.05. These cultures were incubated at a constant temperature shaking incubator at 37 °C, shaking at 120 rpm. The OD_600nm_ values of the cultures were measured at 1 h interval using a spectrophotometer.

### RNA-seq analysis

The experimental operation was performed as previously described with minor modifications [35]. The strains SS HA9801 and Δ*luxS* were cultured in THB medium for 6 h, and harvested at 8000×g at 4 °C for 10 min. Then, the total RNA was extracted with the Trizol Reagent kit (Invitrogen, USA). Three biological replicates were set for each sample, and all samples were sent to Beijing Novogene Co., Ltd. for sequencing by Illumina Hiseq platform. Quality control was performed on the clean reads, and mapping was performed with reference to the SS 05ZYH33 genome. Gene function annotation was performed through the orthologous group (COG) database [36].

### Validation of mRNA-Seq by qRT-PCR

The qRT-qPCR method was used to verify the expression results of mRNA-Seq in the transcriptome. Use Total RNA Extraction Kit (Solarbio, China) to extract total RNA, RNase-free DNase I to remove genomic DNA. The cDNA was amplified using MagicSYBR mix (CoWin Biosciences Co., Ltd., China). The volume of the amplification mixture was set to 20 μl (2× MagicSYBR Mixture 10 μl, each primer of 0.5 μM, cDNA 1 μg, finally add RNase-free water to 20 μl). The PCR reaction conditions were as follows: at 95 °C for 30 s, then at 95 °C for 5 s, and then at 60 °C for 30 s for 40 cycles. Randomly select 6 genes, and use 16S rRNA as internal reference to verify the original data. Table S3 lists all primers.

### Acid stress assay

To assess the sensitivity of SS strains to acid stress conditions, we carried out an acid stress assay as previously described [37], with slight modification. Liquid THB media was prepared with pH values ranging from 3.0 to 7.0 (adjusted with 6 N HCl). Approximately 1 ~ 3 × 10^6^ CFU SS2 bacterial suspension were inoculated at a ratio of 1:10 (v/v) at 37 °C for 12 h or 24 h under aerobic conditions. Growth kinetics of each strain was measured by monitoring OD_600nm_ values under various conditions.

### Fe stress assay

The Fe stress response assay was performed as previously described [38], with some modifications. All strains were sub-cultured at the same original cell density from two subculture in iron restricted THB medium (containing 100 mM iron chelator 2,2′-dipyridyl). Then, transfer all the strains (HA9801, *ΔluxS*, C*ΔluxS*, +*luxS*) grown in iron-restricted THB medium to the same volume of fresh THB medium. Then all the strains cultures were diluted 1: 200 (v/v), and different concentrations of iron ions were added to THB broth, of which Fe^2+^ (0.1, 0.2 and 0.3 mmol/L) and Fe^3+^ (0.1, 0.2 and 0.3 mmol/L). The cultures were incubated at 37 °C with aeration. The above cultures were incubated for 6 h (exponential phase) or 12 h (stable period), and centrifuged at 3000 rpm for 10 min at 4 °C. The bacterial cell pellets were resuspended in the same volume of PBS buffer, and 250 μL of the mixture was added to a 96-well microtiter plate. The assays were performed in triplicate and the OD_600nm_ values were measured.

### Oxidative stress assay

Assessment of the bacterial cells abilities to withstand H_2_O_2_ challenges was determined as previously described [39]. Briefly, SS2 cultures were incubated in THB media until mid-exponential phase (OD_600nm_ ≈ 0.8). For H_2_O_2_ challenge, bacterial cells were prepared similarly, and then incubated in THB containing 58.8 mmol/L H_2_O_2_ for 1 h, 2 h, 3 h or 4 h. After exponentially reasonable dilution, 10 μL of the dilution was spread on THA medium, incubated at 37 °C for 24 h and counted, the bacterial concentration was calculated, and the survival rate was calculated.

### Stress -related gene detection by Quantitative RT-PCR (qRT -PCR)

The qRT-PCR method was used to detect the expression differences of the four strains (HA9801, Δ*luxS*, CΔ*luxS*, +*luxS*) under stress conditions. With reference to the results (Figs. 3, 4 and 5) of the above three stress tests, we chose to perform the qRT-PCR test under conditions where the stress phenotypes are extremely different. In the acid stress test, select the conditions of culturing for 12 h or 24 h at pH = 5, 7; in the iron stress test, select the conditions of culturing for 6 h or 12 h when 0.1 mmol/L Fe (Fe^2+^ or Fe^3+^) is added to the iron-restricted medium; In the oxidative stress, choose the conditions of 1 h or 2 h. The test procedures of total RNA extraction, reverse transcription, and fluorescence quantitative PCR are the same as those in “ Validation of mRNA-Seq by qRT-PCR “ above. Select 6 genes related to stress and two genes related to bacterial morphology, and use 16S rRNA as an internal reference to verify the original data. Table S4 lists all primers.

### Statistical analysis

The Graphad Prism 8.0 software was used to perform statistical analyses for all data. All data points for the experiments, performed in triplicate, were analyzed using the single factor analysis of variance (One-Way ANOVA), where *P* < 0.05 was considered to be statistically significant.

## Supplementary Information

**Additional file 1: Table S1. Transcriptome up-regulated genes and their expression values.**

**Additional file 2: Table S2. Transcriptome down-regulated genes and their expression values.**

**Additional file 3: Table S3.** Primers used for the quantitative RT-PCR analysis.

**Additional file 4: Table S4.** Primers used for the quantitative RT-PCR analysis.

**Additional file 5: Figure S1.** Scanning electron microscopy image of the S. suis biofilms. A: wild-type strain HA9801; B: mutant strain Δ*luxS*; C: complemented strain CΔ*luxS* and; D:overexpression strain *luxS*+. SEM image showing a three-dimensional structure of the biofilm extending vertically from the surface of the membrane. Original magnification was × 5000. Scale: 1 μm.

**Additional file 6: Figure S2.** The growth curve of SS2 wild-type strain HA9801, mutant strain Δ*luxS*, complemented strain CΔ*luxS* and overexpression strain *luxS*+ at 37 °C. Growth was assessed by determination of OD_600nm_ values at the time points indicated. Each time point represents three independent tests.

**Additional file 7: Figure S3.** Detection of six genes expression profiles by qRT-PCR.

**Additional file 8: Figure S4.** Relative expression of environmental fitness genes by *S. suis* HA9801, Δ*luxS*, CΔ*luxS* and *luxS*+ strains. The figure shows that the gene expression level in the HA9801 strain is 100%, and the gene expression in the Δ*luxS*, CΔ*luxS* and *luxS*+ strains were the relative to expression in the HA9801 strain genes. Data from three independent assays are expressed as mean ± SD. *, significantly different at *p* < 0.05; **, significantly different at *p* < 0.01; ***, significantly different at *p* < 0.001.

## Data Availability

All data generated or analyzed during this study are included in this published article and its supplementary information files.
