# Supplementary material for: mRNA-Seq reveals the quorum sensing system luxS gene contributes to the environmental fitness of Streptococcus suis type 2
Source: BMC Microbiol. 2021 Apr 13;21:111. doi: 10.1186/s12866-021-02170-w (PMC8045309; doi:10.1186/s12866-021-02170-w)
Supplement: Supplementary file 3 — Additional file 3: Table S3. Primers used for the quantitative RT-PCR analysis. [file 12866_2021_2170_MOESM3_ESM.docx]

Table S3 Primers used for the quantitative RT-PCR analysis.

| Genes | Primer sequence |
| --- | --- |
| 16S rRNA | GTTGC GAACG GGTGA GTAA |
|  | TCTCA GGTCG GCTAT GTATC G |
| SSU05_2024 | TGGAA ACCAT CCCAA ATGTT |
|  | GTTAA GACCA ACCGC CAAAA |
| SSU05_1111 | TTGGC TGACA AGTCA CGAAG |
|  | GCTGC TTCAC AGAAA GCTCA |
| SSU05_1069 | CATACTGAACAACGACATCTT |
|  | TCCACGAACGAAATAAAAT |
| SSU05_0050 | ATTTG GGATT ACCGT CACA |
|  | AGGGC CTTAT CCGTA ATGCT |
| SSU05_0087 | GCGCT CGTGT ATTGT TGAAA |
|  | CAGAA ACTGA GATGC CACGA |
| SSU05_0302 | GCAAT CCTAA TGCAC CTCGT |
|  | GCATG GTTCC AAGTG GAGTT |
