# Supplementary material for: mRNA-Seq reveals the quorum sensing system luxS gene contributes to the environmental fitness of Streptococcus suis type 2
Source: BMC Microbiol. 2021 Apr 13;21:111. doi: 10.1186/s12866-021-02170-w (PMC8045309; doi:10.1186/s12866-021-02170-w)
Supplement: Supplementary file 4 — Additional file 4: Table S4. Primers used for the quantitative RT-PCR analysis. [file 12866_2021_2170_MOESM4_ESM.docx]

Table S4 Primers used for the quantitative RT-PCR analysis.

| Genes | Primer sequence |
| --- | --- |
| 16S rRNA | GTTGC GAACG GGTGA GTAA |
|  | TCTCA GGTCG GCTAT GTATC G |
| SSU05_0760 | TGAATTAGCCCGTCTTGAA |
|  | CTTTGGTTGGCAATGTTGA |
| SSU05_0761 | TCAAAGGTTTCGTGAGGTA |
|  | GTGATGTCAACATTGCCA |
| SSU05_1677 | TGAAATAAGTAGCCTGACC |
|  | CTTTGATGAAATCGTTGAA |
| SSU05_0650 | AAAGCGACCATAGGAGACC |
|  | GGACAAGCCATTTCACAAC |
| SSU05_2171 | GGGTATTGCTGTCCTTGTT |
|  | ACGGATGTAGCCTTGGTAG |
| SSU05_1508 | GACGCCTGATGTATCCCTA |
|  | CTACGCCTCCATTCCTTCT |
| SSU05_1111 | TTGGC TGACA AGTCA CGAAG |
|  | GCTGC TTCAC AGAAA GCTCA |
| SSU05_1069 | CATACTGAACAACGACATCTT |
|  | TCCACGAACGAAATAAAAT |
