# Supplementary figures and images for: mRNA-Seq reveals the quorum sensing system luxS gene contributes to the environmental fitness of Streptococcus suis type 2
Source: BMC Microbiol. 2021 Apr 13;21:111. doi: 10.1186/s12866-021-02170-w (PMC8045309; doi:10.1186/s12866-021-02170-w)

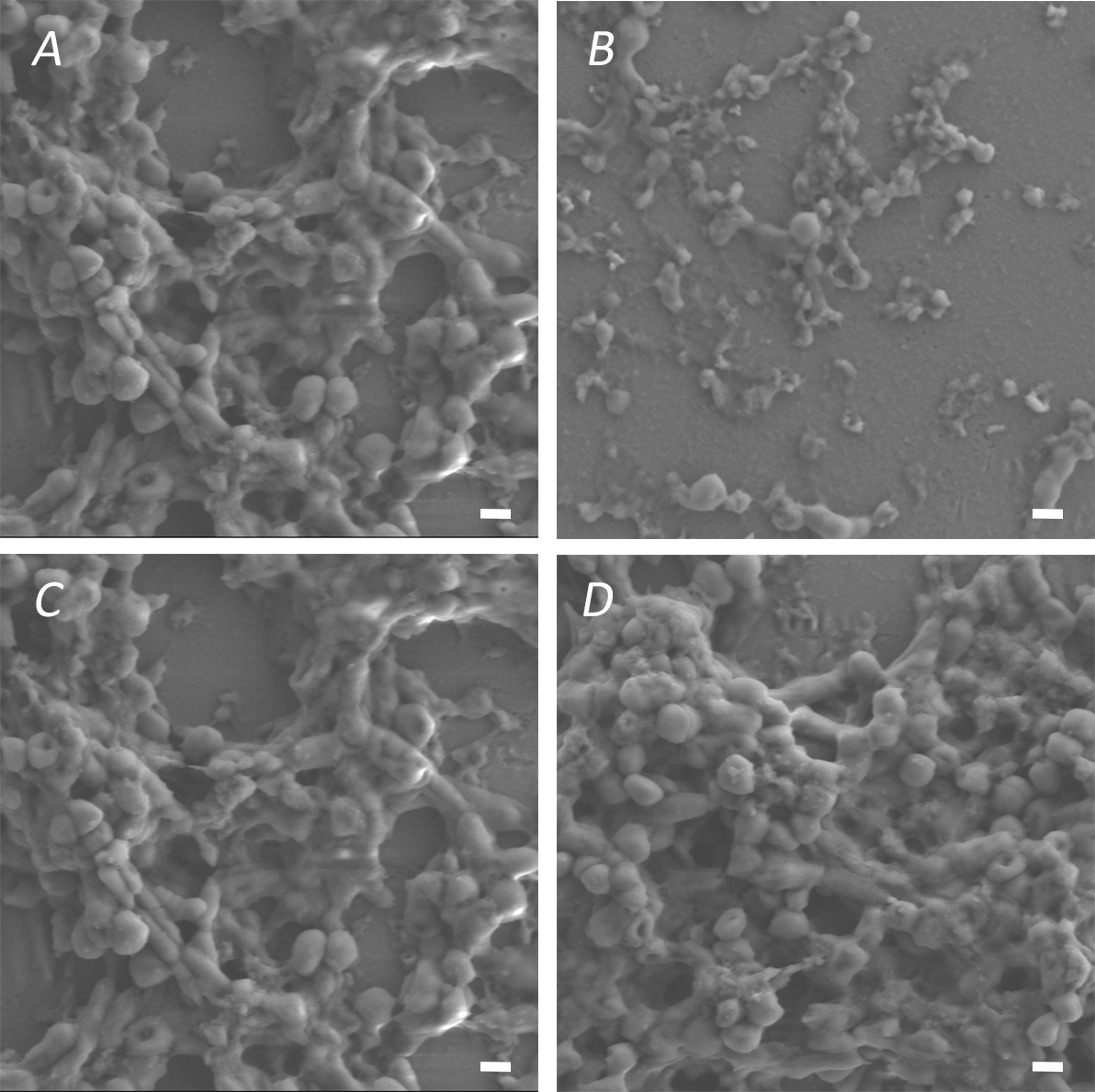

Supplement: Supplementary file 5 — Additional file 5: Figure S1. Scanning electron microscopy image of the S. suis biofilms. A: wild-type strain HA9801; B: mutant strain ΔluxS; C: complemented strain CΔluxS and; D:overexpression strain luxS+. SEM image showing a three-dimensional structure of the biofilm extending vertically from the surface of the membrane. Original magnification was × 5000. Scale: 1 μm. [file 12866_2021_2170_MOESM5_ESM.jpg]

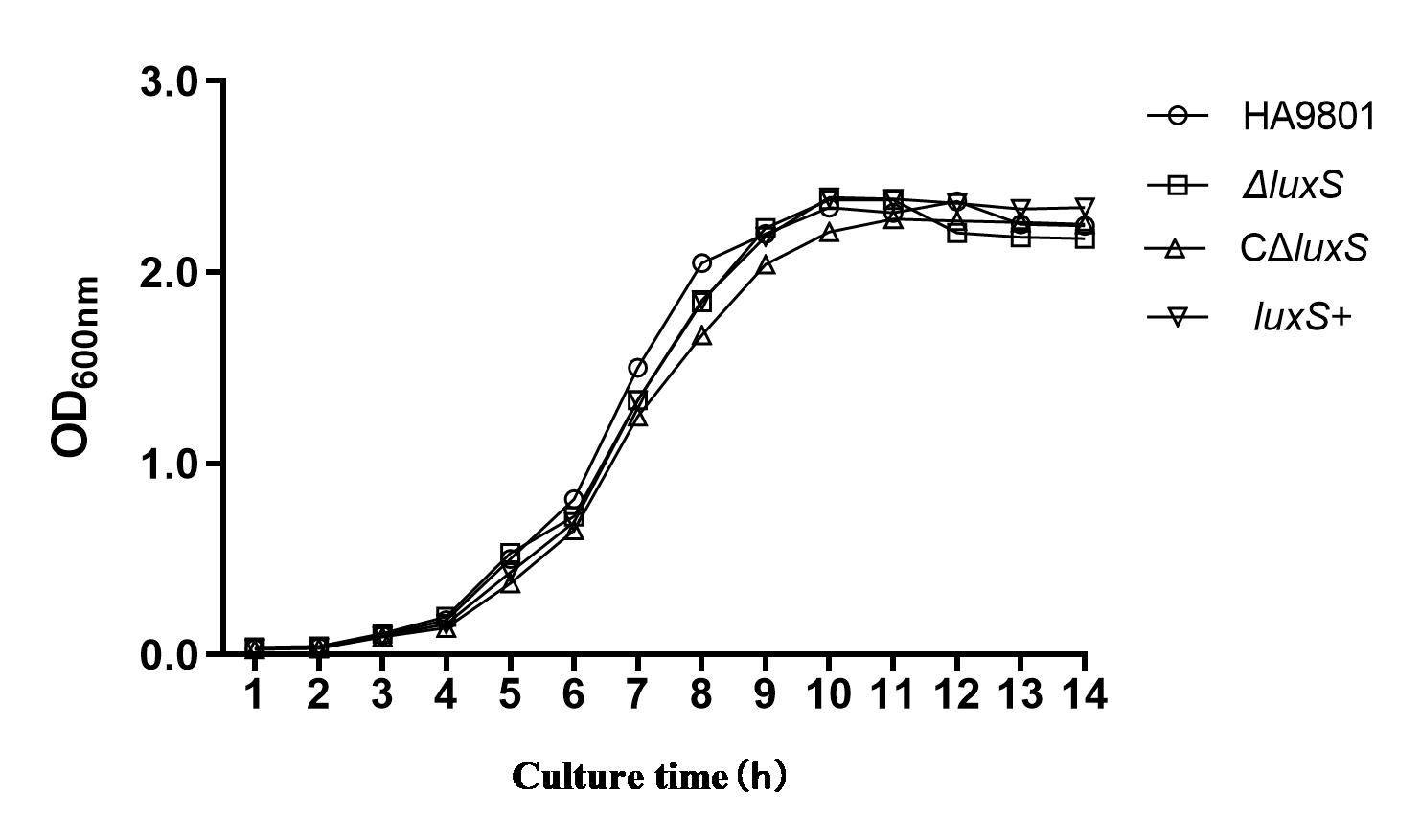

Supplement: Supplementary file 6 — Additional file 6: Figure S2. The growth curve of SS2 wild-type strain HA9801, mutant strain ΔluxS, complemented strain CΔluxS and overexpression strain luxS+ at 37 °C. Growth was assessed by determination of OD600nm values at the time points indicated. Each time point represents three independent tests. [file 12866_2021_2170_MOESM6_ESM.jpg]

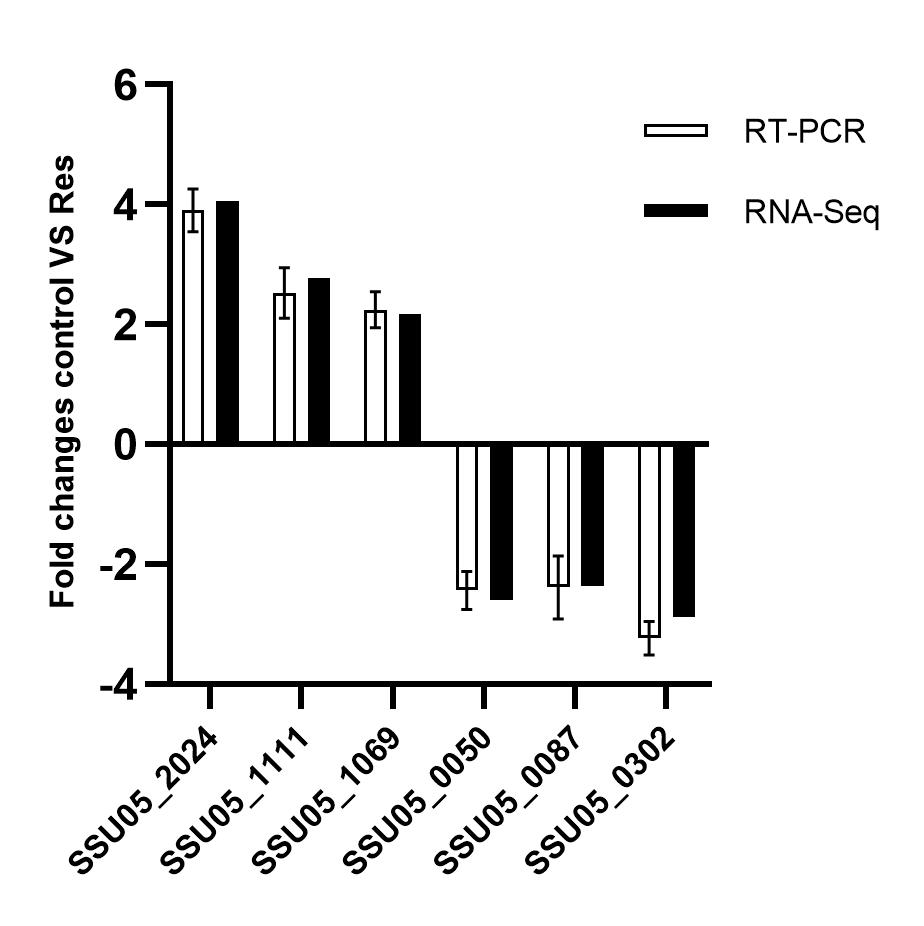

Supplement: Supplementary file 7 — Additional file 7: Figure S3. Detection of six genes expression profiles by qRT-PCR. [file 12866_2021_2170_MOESM7_ESM.jpg]

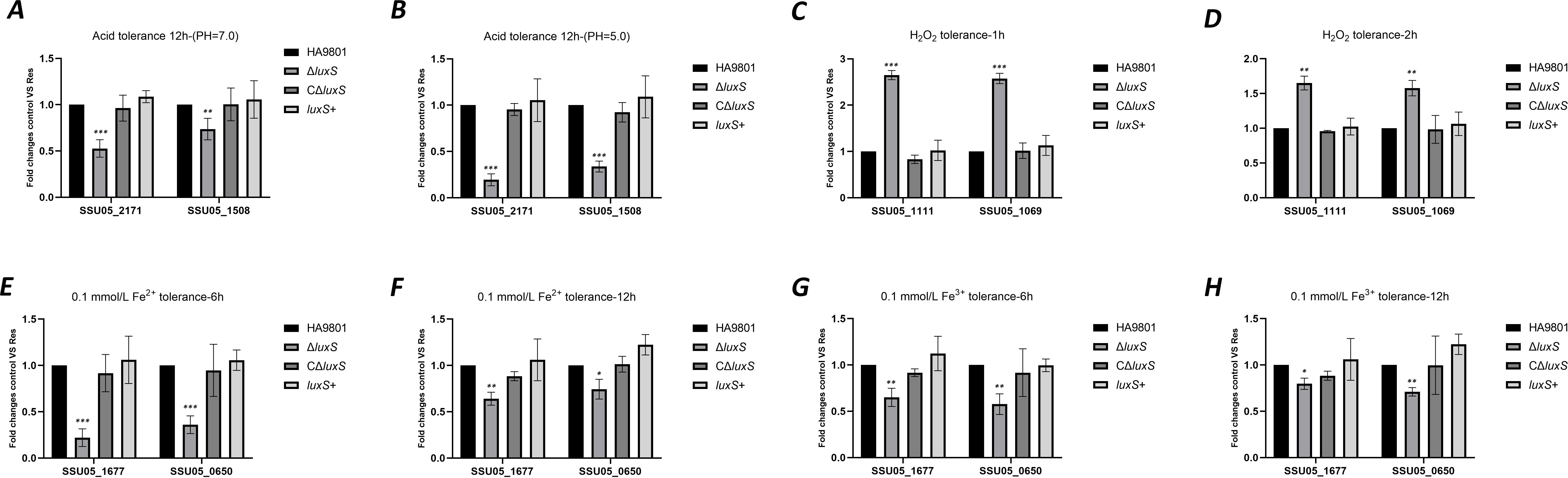

Supplement: Supplementary file 8 — Additional file 8: Figure S4. Relative expression of environmental fitness genes by S. suis HA9801, ΔluxS, CΔluxS and luxS+ strains. The figure shows that the gene expression level in the HA9801 strain is 100%, and the gene expression in the ΔluxS, CΔluxS and luxS+ strains were the relative to expression in the HA9801 strain genes. Data from three independent assays are expressed as mean ± SD. *, significantly different at p < 0.05; **, significantly different at p < 0.01; ***, significantly different at p < 0.001. [file 12866_2021_2170_MOESM8_ESM.jpg]
